# Factors influencing perseverance in teaching Chinese martial arts abroad: a self-determination theory perspective among international instructors

**DOI:** 10.3389/fpsyg.2024.1391207

**Published:** 2024-04-30

**Authors:** Xueying Cao, Hui Lyu

**Affiliations:** ^1^Faculty of Sports Science, Ningbo University, Ningbo, China; ^2^Ningbo Innovation Center, Zhejiang University, Ningbo, China

**Keywords:** self-determination theory, Chinese martial arts (CMAs), instructor persistence, autonomous motivation, amotivation, questionnaire, structural equation modeling (SEM)

## Abstract

**Background:**

The global dissemination of Chinese martial arts (CMAs), transcends mere physical activity; it represents a cultural and philosophical journey that contributes to enhanced psychological well-being. Capturing international attention, CMAs have engendered a network of global instructors committed to their cross-cultural teaching. However, the narrative of CMAs across the globe is incomplete without understanding the psychological factors that fuel the perseverance of these international instructors. Their relentless commitment, motivated by factors beyond the cultural and geographical barriers, poses a unique question: What motivates these instructors to persist in teaching CMAs in the face of such challenges? The study aims to uncover the key motivational mechanisms that influence the perseverance of international CMAs instructors in their teaching endeavors.

**Methods:**

Employing Self-Determination Theory, 147 international CMAs instructors completed the Motivation for Teaching CMAs Scale, Perceived Belonging Scale, and Perseverance in Teaching CMAs Scale. SPSS 20.0 was utilized for conducting descriptive statistics, common method bias tests, and correlation analyses. Structural equation modeling was performed using AMOS 26.0.

**Results:**

Autonomous Motivation positively affected perseverance in teaching CMAs (*β* = 0.369, *b* = 0.465, *t* = 4.232, *p* < 0.001). In contrast, Amotivation negatively affected perseverance (*β* = −0.323, *b* = −0.382, *t* = −3.561, *p* < 0.001). Neither Controlled Motivation nor Sense of Belonging significantly affected perseverance. The model explained 27.9% of the variance in perseverance, offering insights into the motivational mechanisms influencing international CMAs instructors.

**Conclusion:**

This study concludes that the perseverance of international instructors in teaching CMAs is primarily driven by overcoming amotivation and fostering autonomous motivation, rather than short-term internal or external incentives, which appear ineffective. Additionally, sense of belonging to their CMA school does not significantly influence their perseverance, potentially due to the diverse cultural backgrounds of the instructors surveyed. The findings suggest that by enhancing the recognition and acceptance of CMAs’ core philosophies and values, aligning teaching practices with personal and cultural values, and fostering a profound passion for CMAs, international instructors could boost their autonomous motivation, which is crucial for their sustained commitment in promoting CMAs globally.

## 1 Introduction

The global dissemination of Chinese martial arts (CMAs), known as Wushu, transcends the mere transmission of physical skills, embodying a rich tapestry of cultural heritage, philosophy, and psychological well-being (Jennings et al., 2010; Guo et al., 2014; Kee, 2019). CMAs, a term that encapsulates a variety of styles and practices originating from China, has not only been a cornerstone of Chinese cultural identity but has also captivated the interest of enthusiasts and practitioners worldwide (Farrer and Whalen-Bridge, 2011; Lau, 2022). This international appeal has led to the emergence of a dedicated cadre of instructors who play a pivotal role in the cross-cultural exchange and teaching of CMAs outside its homeland.

Teaching CMAs abroad encompasses more than mere cultural dissemination; it represents a practice that underscores the harmony between mind and body, fostering psychological resilience, discipline, and well-being (Jennings, 2014). As CMAs continue to weave their narrative across the globe, grasping the psychological drivers that underpin the perseverance of international instructors becomes paramount. These individuals serve not only as ambassadors of CMAs but also as custodians of its cultural and psychological significance. Their dedication to teaching in environments markedly different from the art’s origins magnifies the critical role of motivation behind their commitment. This commitment raises a compelling inquiry into the motivations or forces that sustain their teaching endeavors despite the challenges posed by cultural and geographical dislocation.

While existing literature has extensively explored the technical training aspects and cultural values of CMAs, there appears to be a significant gap in understanding the motivational dynamics of international CMAs instructors. Research highlighting the biomechanical and physiological benefits of CMAs practice, especially its positive effects on physical health and skill development (Lip et al., 2015; Wang et al., 2024). Concurrently, the work of Guo et al. (2014) has elucidated the cultural and historical significance of CMAs, underscoring its role in promoting Chinese heritage globally. However, these inquiries have predominantly concentrated on the art form itself, with limited attention given to the instructors who are pivotal in its cross-cultural transmission.

Specifically, the psychological resilience and motivation required for instructors to persist in teaching CMAs, especially in environments vastly different from their origins, remain poorly understood. Although some studies have noted the pedagogical challenges CMAs instructors face in non-native settings (Sun and Wang, 2011; Yuan, 2017; Lu and Li, 2019), the detailed investigation into the motivational factors that empower these instructors to overcome such challenges has yet to be conducted. This oversight highlights a critical research gap: the lack of comprehensive understanding of what drives international CMAs instructors to continue their teaching endeavors despite the obstacles they face.

This study aims to fill this gap by applying Self-Determination Theory (SDT) to explore the intrinsic and extrinsic motivational factors influencing the perseverance of international CMAs instructors. By shifting the focus from the art form to the educators behind its global dissemination, this research contributes to a more nuanced understanding of the psychological factors that support the sustained teaching of CMAs abroad.

Self-Determination Theory (SDT), is a widely recognized framework for understanding human motivation and psychological well-being. Developed by Deci and Ryan (1985), SDT distinguishes between different types of motivation based on the degree to which they are self-determined or autonomous. Based on the nature of motivation, SDT categorizes motivation into three major types: autonomous motivation, controlled motivation, and amotivation. Autonomous motivation further encompasses three specific forms of motivation: intrinsic motivation integrated regulation, and identified regulation; controlled motivation includes two specific forms: introjected regulation and external regulation (Vansteenkiste and Sheldon, 2006; Ratelle et al., 2007). Intrinsic motivation represents the highest degree of autonomy in motivation, referring to behaviors that are driven entirely by internal interest and pleasure, unrelated to any external rewards. Integrated regulation is a form of motivation with a high degree of autonomy, where external objectives are accepted as personally important goals, and these goals are integrated into one’s core values and beliefs. Identified regulation refers to a more autonomous form of motivation, where individuals recognize and accept the intrinsic value of a behavior, incorporating it into their sense of self. Introjected regulation refers to behavior taken to avoid feelings of guilt or self-reproach, with actions controlled by internal factors. External regulation is the form of motivation with the highest level of control, where people’s performance and behavior are aimed at obtaining rewards or avoiding punishments, controlled by external factors. Amotivation describes a state in which an individual is either not motivated to act or acts without intentionality (Deci and Ryan, 2000; Ryan and Deci, 2000).

In the domain of sports psychology, SDT has been applied to understand the motivation behind sports participation, coaching behaviors, and athletes’ performance and persistence (Ratelle et al., 2007; Reynders et al., 2019). Despite this, the application of SDT in the context of international CMAs teaching remains scantily explored. In light of this, the current study aims to fill this gap by exploring the motivational factors affecting the persistence of international CMAs instructors in overseas teaching, thereby further deepening the understanding of psychological dynamics in the context of cultural and educational exchanges.

**Figure 1:**
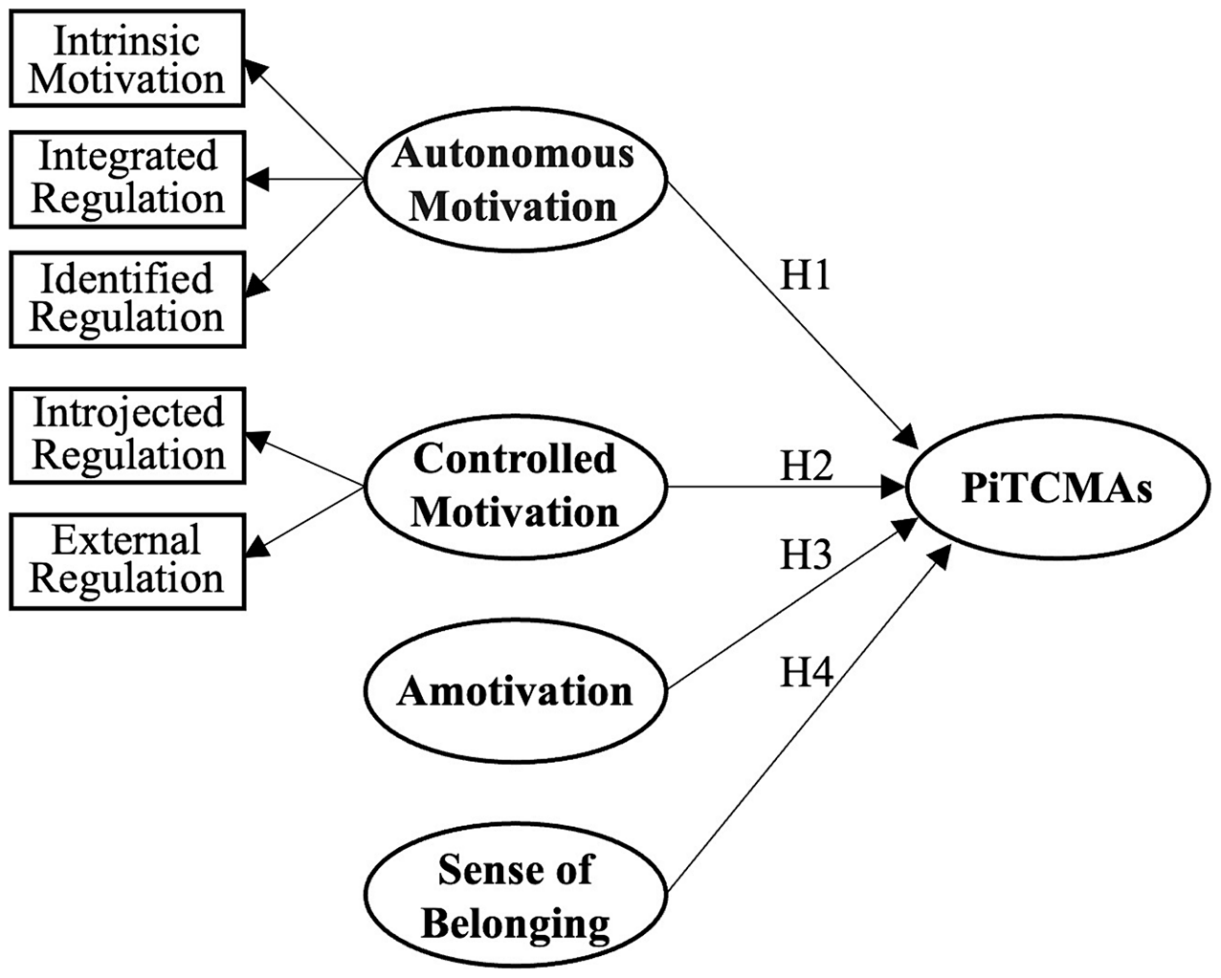
Research Model. Note: PiTCMA – Perseverance in Teaching Chinese Martial Arts.

Research indicates that autonomous motivation, which includes intrinsic motivation, integrated regulation, and identified regulation, plays a crucial role in the persistence of behaviors (Ryan et al., 1997; Deci and Ryan, 2008; Keshtidar and Behzadnia, 2017). Identified regulation, a subcomponent of autonomous motivation, has been shown to be particularly highly related to persistence (Howard et al., 2021). The more an individual’s behavior regulation aligns with autonomous motivation, the higher their willingness to engage, the level of initiative and involvement, and the duration of persistence (Oman and McAuley, 1993; Levesque et al., 2007; Dong and Mao, 2020). Based on these findings, this study proposes the following hypotheses (see Figure 1):

*H1*: Autonomous motivation, encompassing intrinsic motivation, integrated regulation, and identified regulation, will positively influence perseverance in teaching CMAs.

Controlled motivation, which encompasses introjected regulation and external regulation, refers to the type of motivation that is subject to external forces. Introjected regulation is a form of controlled motivation where behavior is regulated by internal pressures like self-esteem and pride, or by the desire to avoid guilt and self-reproach, which is considered to have lower voluntariness than autonomous motivation (Pelletier et al., 2001). External regulation, another component of controlled motivation, is influenced by external rewards or punishments and lacks interaction with intrinsic motivation (Vlachopoulos and Karageorghis, 2005). Howard et al. (2021) found that external regulation was not associated with persistence. Further, Keshtidar and Behzadnia (2017) found that controlled motivation does not significantly predict the intention to continue sport in the future. Given these insights, this study hypothesizes that:

*H2*: Controlled motivation, encompassing both introjected and external regulation, will not significantly affect the perseverance of instructors in teaching CMAs.

Furthermore, individuals lacking motivation or being completely amotivated are more likely to give up halfway (Vallerand et al., 1997). Higher levels of amotivation have been linked to increased sport dropout (Calvo et al., 2010). Consequently, it is hypothesized:

*H3*: Amotivation will negatively impact instructors’ perseverance in teaching CMAs.

Incorporating the Self-Determination Theory (SDT), belongingness is identified as a fundamental psychological need, alongside autonomy and competence, that underpins human motivation and well-being (Deci and Ryan, 2000). The sense of belonging, or relatedness, is crucial for motivating self-determined behaviors, driving individuals to seek connections and nurture relationships, thereby fostering a sense of unity and mutual care within groups (Deci and Ryan, 1991). This theoretical perspective underscores the relational need for individuals to feel connected and valued within their social contexts. Carron et al. (1988) support this by demonstrating that team cohesion, which encompasses a strong sense of belonging, significantly correlates with sustained exercise behavior and adherence. Therefore, acknowledging the pivotal role of belonging within SDT’s framework,

*H4*: A sense of belonging will significantly enhance the perseverance of instructors in teaching CMAs.

Although Self-Determination Theory (SDT) has been widely applied to understand motivation in sports participation, coaching behaviors, and athletes’ performance and persistence (Ratelle et al., 2007; Reynders et al., 2019), its application in the context of international teaching of CMAs remains scantily explored. The current study aims to fill this gap by exploring the motivational factors affecting the persistence of international CMA instructors in their overseas teaching, thereby deepening our understanding of psychological dynamics within the context of cultural and educational exchanges. Specifically, this research focuses on the effects of autonomous motivation, controlled motivation, amotivation, and a sense of belonging on instructors’ perseverance in teaching, aspects not thoroughly discussed in existing literature. By applying the theoretical framework of SDT to the practice of international CMAs teaching, this study not only unveils key psychological mechanisms driving instructors’ sustained teaching commitment but also provides theoretical and practical guidance for promoting the global dissemination of CMAs. Therefore, this research contributes new perspectives to the field of cultural sports psychology, highlighting the significance of psychological well-being in the international protection and promotion of intangible cultural heritage.

## 2 Materials and methods

### 2.1 Participants

This study initially employed Apify’s Google Maps Scraper service to harvest information on 16,382 Chinese Martial Arts (CMAs) dojos. Of these, 11,894 entries included website information. After removing duplicates—accounting for the fact that some dojos shared the same website, which led to repetitive website entries—the data set was refined to 8,929 unique website URLs. Subsequently, the study utilized the Octoparse Web Scraping Tool to methodically extract email addresses from the homepage of each of these 8,929 websites, ultimately gathering 2,374 email addresses.

Following data collection, a survey questionnaire, created using Google Forms, was distributed to the 2,374 acquired email addresses through a targeted “BCC” (blind carbon copy) email campaign. To further maximize information collection, the survey was also disseminated within several Facebook groups centered on Chinese martial arts, such as Chinese Martial Art & Kung-Fu Club, Traditional Chinese Martial Arts Community, Chinese Martial Arts, Wing Chun Forum, Hakka Kung Fu, Wushu Stars, and Monkey Steals Peach. These groups were approached with prior consent from their administrators to post the survey invitation. This approach resulted in 156 international CMAs instructors completing the questionnaire.

The collected questionnaires underwent a rigorous screening process based on predetermined criteria, which included checking for patterned responses, inconsistencies in answers, and duplicate submissions. Following this scrutiny, a total of 147 questionnaires were deemed valid, achieving a valid response rate of 94.2%.

#### 2.1.1 Demographic characteristics

Table 1 shows the basic information of the survey participants. Ages were primarily above 35 years, with 26.5% between 35 and 44, 32.0% between 45 and 54, and 28.6% over 55. The sample was predominantly male (91.5%), likely due to the masculine nature of CMAs, resulting in higher male participation and making data from female instructors abroad more difficult to obtain. Additionally, the CMAs instructors surveyed had a high duration of practice, with 94.5% practicing CMAs for more than 10 years, and 66.7% teaching CMAs for over a decade. A total of 59.9% had a bachelor’s degree or higher, possibly influenced by the educational level during the collection of non-English speaking countries’ questionnaires, but consequently, most data came from English-speaking countries. The data showed that 12.2% of participants were ethnic Chinese. This indicates that the international CMAs instructors studied were predominantly non-Chinese, potentially providing strong evidence for the reasons non-Chinese individuals persist in teaching CMAs.

**Table 1:** Demographic characteristics of the study group (*N* = 147).

Variables	Distribution	Percent (%)	Variables	Distribution	Percent (%)
Gender	Male	91.5	Age	18–24	2.0
	Female	8.5		25–34	10.9
Ethnic Chinese	Yes	12.2		35–44	26.5
	No	87.8		45–54	32.0
Education level	Bachelor’s Degree	21.8		55–64	21.8
	Graduate Degree	30.6		65–74	6.8
	High School	15.6	Years of CMA Practice	≤10 years	5.5
	Professional Degree	7.5		11–20 years	27.2
	Other	24.5		21–30 years	29.9
Nationality	Brazil	15.1		31–40 years	22.4
	United Kingdom	15.1		>40 years	15.0
	United States	14.4	Years of CMA Teaching	≤5 years	17.0
	Germany	7.5		6–10 years	16.3
	Italy	6.2		11–20 years	34.0
	Canada	4.8		21–30 years	17.7
	Other	36.9		>30 years	15.0

#### 2.1.2 CMAs content selection

The surveyed CMAs instructors taught a variety of styles, with Yang Style Tai Chi (34.7%) and Wing Chun (26.5%) being the most popular. Moreover, these instructors mainly disseminated the following contents:

First, combat techniques are considered the most important aspect of teaching CMAs by respondents (97.3%), reflecting the essence of combat in CMAs. Among them, 69.4% of instructors teach according to a systematic curriculum (grading), while some do not follow a grading system, believing that teaching authentic fighting techniques is most crucial;

Second, 91.2% of the respondents believe it is essential for students to understand the historical background and lineage of the CMAs they learn. This not only enhances the students’ or disciples’ understanding of CMAs but also fosters a sense of pride, belonging, and cohesion;

Third, 90.5% of respondents share the cultural and philosophical foundations of CMAs, such as Yin and Yang, Ba Gua, Buddhism, and Taoism, during their teaching. Some instructors view CMAs as a lifestyle, integrating its culture and philosophy into daily practice. As these instructors themselves benefit from the culture and philosophy of CMAs, they particularly emphasize these aspects in their teaching;

Fourth, 83.7% of respondents believe that Wu De (martial virtue) is an important teaching content. They argue that a clear set of values, such as respect, self-discipline, perseverance, and integrity, should be established in the dissemination of CMAs. This way, one can “share benevolence, the heart of Kung Fu.” Additionally, 76.2% of respondents emphasized the importance of Wu Li (martial etiquette), showcasing the relationship between Wu De and Wu Li and reflecting the combination of moral cognition and moral practice in CMAs (Lin and Cao, 2020). Lastly, some respondents mentioned teaching some martial arts terms in Chinese, such as titles within the martial family and names of movements, during the dissemination process.

### 2.2 Instruments

This study incorporated three scales: Motivation for Teaching CMAs Scale, Perceived Belonging Scale and Perseverance in Teaching CMAs Scale. To ensure the reliability and validity of the measurement tools, this research primarily utilized scales that have been previously employed in studies, which were then modified according to the research objectives to serve as empirical tools.

- Motivation for Teaching CMAs Scale. The Motivation for Teaching CMAs Scale, inspired by Self-Determination Theory (Deci and Ryan, 1980), incorporates elements from several established scales: the Work Tasks Motivation Scale for Teachers (WTMST) (Fernet et al., 2008), Work Extrinsic and Intrinsic Motivation Scale (WEIMS) (Tremblay et al., 2009), Motivation at Work Scale (MAWS) (Gagné et al., 2010), and the Multidimensional Work Motivation Scale (MWMS) (Gagné et al., 2015). This comprehensive scale categorizes motivation for teaching CMAs into six dimensions: Intrinsic Motivation, Integrated Regulation, Identified Regulation, Introjected Regulation, External Regulation, and Amotivation, with each dimension comprising three items for a total of 18 items. It has demonstrated high reliability with Cronbach’s alpha coefficients above 0.80 for all dimensions and its structural validity has been validated through Structural Equation Modeling, reflecting a high fit (Gagné et al., 2015). Items on this scale are rated using a 7-point Likert scale that ranges from 1 (Not at all) to 7 (Exactly), allowing for a nuanced assessment of instructors’ motivations for teaching CMAs.
- Perceived Belonging Scale. Belongingness refers to the instructors’ sense of subordination to, identification with, and maintenance of their affiliation with their mentorship or group. Referencing the Perceived Belonging Scale (PBS), this scale is constructed based on Self-Determination Theory and utilizes 11 items to measure the communicators’ perceived belonging. The scale’s Cronbach’s alpha coefficients is above 0.70, and its construct validity has been verified through Structural Equation Modeling, showing a high level of fit (Allen, 2006). Items are measured using a Likert 7-point scale, ranging from 1 (Disagree strongly) to 7 (Agree strongly).
- Perseverance in Teaching CMAs Scale. This scale measures the extent to which instructors persist in their efforts to spread CMAs amidst challenges and obstacles. The perseverance scale developed by Liu et al. (2011) has an alpha coefficient of 0.85, indicating good fit. After revising this scale to suit Perseverance in Teaching CMAs, it was translated into English through a back-translation method by two bilingual translators (Brislin, 1980). Items are measured using a Likert 5-point scale, with options ranging from 1 (Strongly disagree) to 5 (Strongly agree).

### 2.3 Analysis

The study utilized SPSS 20.0 and the Structural Equation Modeling (SEM) software AMOS 26.0 to conduct empirical analysis following these steps: First, employing a single-factor method for common method bias test; Second, using confirmatory factor analysis to test reliability and validity as well as the fit of the measurement model; Third, exploring the relationships between variables through correlation analysis; Fourth, assessing the overall fit of the structural model; Fifth, revising and interpreting the results of the model fit.

Additionally, the data underwent the following processes:

(1) Scale conversion. As the majority of scales used in this study were 7-point scales, the 5-point scale, was uniformly converted to 7-point scales for data analysis. The conversion formula is: $Y=\left(B-A\right)\times \frac{x-a}{b-a}+A$ , where *Y* is the function of the converted scale, *X* is the function of the scale used in the original questionnaire, *a* and *b* are the minimum and maximum values of the original scale, and *A* and *B* are the minimum and maximum values of the converted scale, respectively.

(2) Removal of outliers. This study excluded 3 outliers that exceeded the Mahalanobis distance, leaving 144 data entries.

(3) Item parceling. SEM analysis typically requires a sample to observed variable ratio of at least 10:1 (Thompson, 2000). Given the challenge of obtaining overseas sample data, the small sample size, and the complexity of the model, this study, based on the reliability and validity tests of the scales’ latent variables, parceled single-dimensional, homogeneous latent variables. For instance, under the second-order model of autonomous motivation, three first-order indicators were separately parceled, such as Q30a = (Q30a_1 + Q30a_2 + Q30a_3)/3. To further address these challenges, the high-medium loading method was employed for item parceling (Wu and Wen, 2011). This method involves arranging items based on their factor loadings from highest to lowest and then creating groups that each contain a high-loading, a medium-loading, and a low-loading item, thereby ensuring a balanced representation of item loadings within each parcel. This balanced approach helps in maintaining the integrity and variability of the underlying construct across the parcels. Applying this method, the Perceived Belonging Scale’s 8 items were parceled into 3 groups, with the first group including items Q29_11, Q29_5, Q29_10; the second group containing items Q29_6, Q29_7, Q29_8; and the third group including items Q29_4, Q29_2. Similarly, the Perseverance in Teaching CMAs Scale’s 5 items were parceled into 3 groups, with the first group containing items Q8_1, Q8_6; the second group containing items Q8_2, Q8_5; and the third group containing item Q8_3. Through this method, we ensured that each parcel accurately represents the construct’s range of factor loadings, thereby facilitating a more reliable analysis within the constraints of our study’s sample size and structural complexity.

## 3 Results

### 3.1 Test for common method bias

Given that all data were self-reported by CMAs instructors, the study first employed Harman’s single-factor test to examine common method bias. The exploratory factor analysis with rotation identified six factors with eigenvalues greater than 1, where the largest factor accounted for 32.765% of the variance. These results are in line with the criteria proposed by Podsakoff et al. (2003), where more than one factor with eigenvalues greater than 1 and the largest factor’s variance explanation being less than 40% indicate that severe common method bias is not present in this study.

### 3.2 Reliability and validity test and analysis of the fit of the measurement model

This study analyzes the correspondence between measurement factors and items through Confirmatory Factor Analysis (CFA) (see Table 2).

**Table 2:** Summary of confirmatory factor analysis for each factor in the research model (*N* = 144).

Factor	Item	Model parameter estimates				Convergent validity				Goodness-of-fit indexes					
		UFL	S.E.	*t*	*P*	SFL	SMC	C.R.	AVE	*χ* ^2^	df	*χ*^2^/df	GFI	AGFI	RMSEA
Intrinsic motivation (*α* = 0.910)	Q30a_1	1.000				0.925	0.856	0.916	0.785	0.000	0	–	–	–	–
	Q30a_2	0.906	0.068	13.388	^***^	0.857	0.734								
	Q30a_3	0.894	0.075	11.987	^***^	0.875	0.766								
Integrated regulation (*α* = 0.942)	Q30b_1	1.000				0.930	0.865	0.939	0.838	0.000	0	–	–	–	–
	Q30b_2	1.073	0.053	20.063	^***^	0.946	0.895								
	Q30b_3	1.069	0.065	16.469	^***^	0.868	0.753								
Identified regulation (*α* = 0.733)	Q30c_1	1.000				0.575	0.331	0.825	0.618	0.000	0	–	–	–	–
	Q30c_2	1.098	0.157	6.995	^***^	0.844	0.712								
	Q30c_3	0.888	0.131	6.797	^***^	0.901	0.812								
Introjected regulation (*α* = 0.835)	Q30d_1	1.000				0.803	0.645	0.855	0.670	0.000	0	–	–	–	–
	Q30d_2	1.071	0.108	9.942	^***^	0.990	0.980								
	Q30d_3	0.707	0.087	8.171	^***^	0.621	0.386								
External regulation (*α* = 0.923)	Q30e_1	1.000				0.882	0.778	0.922	0.798	0.000	0	–	–	–	–
	Q30e_2	0.999	0.067	14.984	^***^	0.897	0.805								
	Q30e_3	0.986	0.066	15.036	^***^	0.900	0.810								
Amotivation (*α* = 0.843)	Q30f_1	1.000				0.646	0.417	0.860	0.677	0.000	0	–	–	–	–
	Q30f_2	1.233	0.148	8.344	^***^	0.942	0.887								
	Q30f_3	1.057	0.124	8.548	^***^	0.853	0.728								
Sense of belonging (*α* = 0.932)	Q29_2	1.000				0.759	0.576	0.937	0.654	29.185	20	1.459	0.955	0.919	0.056
	Q29_4	1.131	0.105	10.798	^***^	0.831	0.691								
	Q29_5	1.145	0.110	10.422	^***^	0.807	0.651								
	Q29_6	1.103	0.102	10.811	^***^	0.832	0.692								
	Q29_7	1.140	0.109	10.509	^***^	0.813	0.661								
	Q29_8	1.011	0.113	8.956	^***^	0.709	0.503								
	Q29_10	1.052	0.110	9.592	^***^	0.753	0.567								
	Q29_11	1.154	0.092	12.559	^***^	0.942	0.887								
PiTCMA (*α* = 0.826)	Q8_1	1.000				0.879	0.773	0.837	0.517	11.013	5	2.203	0.970	0.910	0.092
	Q8_2	1.052	0.087	12.025	^***^	0.868	0.753								
	Q8_3	0.852	0.100	8.482	^***^	0.654	0.428								
	Q8_5	0.714	0.117	6.090	^***^	0.499	0.249								
	Q8_6	0.739	0.094	7.864	^***^	0.617	0.381								

UFL, unstandardized factor loadings; S.E., standard error; SFL, standardized factor loadings; SMC, squared multiple correlations; C.R., composite reliability; AVE, average variance extracted; df, degrees of freedom; GFI, goodness of fit index; AGFI, adjusted goodness of fit index; RMSEA, root mean square error of approximation; PiTCMA, persistence in teaching Chinese martial arts; ^***^p < 0.001.

(1) Measurement Model Fit. Items within each latent variable with standardized factor loadings below 0.4 were deleted (Hair et al., 2014); model fit indices were checked, and items causing excessively high chi-square values due to residual correlations, indicating item similarity, were removed (Landis et al., 2009). Ultimately, items 1, 3, and 9 from the Perceived Belonging Scale (Q29) and item 4 from the Perseverance in Teaching CMAs Scale (Q8) were deleted, achieving a satisfactory model fit for each latent variable, with fit indices meeting the criteria of *χ*^2^/df < 3.0, AGFI >0.90, CFI > 0.90, RMSEA <0.08 (Hair et al., 2014). Latent variables not annotated with fit indices in the table are due to having only three items, constituting a just-identified model, where the number of data points matches the number of parameters to be estimated in the model, resulting in zero degrees of freedom, also known as a saturated model.

(2) Internal Consistency Coefficient (α). This value is a commonly used index for testing reliability, with the formula: $\alpha =\frac{K}{K-1}\left(1-\frac{\sum S_{i}^{2}}{S^{2}}\right)$ , where *K* is the number of items in the scale, $\sum S_{i}^{2}$ is the total variance of the scale items, and $S^{2}$ is the variance of the total score of the scale items. The *α* coefficient ranges from 0 to 1, with DeVellis (2017) suggesting that values between 0.65 and 0.70 are the minimum acceptable; values between 0.70 and 0.80 are quite good; values between 0.80 and 0.90 are very good. All latent variables in this study had *α* coefficient values above 0.70, indicating quite good internal consistency.

(3) Convergent Validity is represented by the Average Variance Extracted (AVE), which can be calculated using the formula: $AVE=\frac{\left(\sum \lambda^{2}\right)}{\left(\left(\sum \lambda^{2}\right)+\sum \theta \right)}$ , where *λ* represents the standardized factor loadings of the observed variables on the latent variable, and *θ* represents the error variance of the indicator variables. AVE reflects the extent to which a latent variable construct can explain the variance of its indicator variables. Higher AVE values indicate higher reliability and convergent validity of the construct. Fornell and Larcker (1981b) consider values between 0.36 and 0.5 as the minimum acceptable, and values above 0.5 as ideal. All latent variables in this study had AVE values above 0.5, indicating good convergent validity.

(4) Composite Reliability (CR). This value can be calculated using the formula: $CR=\frac{{\left(\sum \lambda \right)}^{2}}{\left({\left(\sum \lambda \right)}^{2}+\sum \theta \right)}$ , where *λ* represents the standardized factor loadings of the observed variables on the latent variable, and *θ* represents the error variance of the indicator variables. CR indicates whether all items within each latent variable consistently explain that latent variable. Fornell and Larcker (1981a) suggest that a CR value above 0.6 indicates good composite reliability. All latent variables in this study had CR values above 0.6, indicating good composite reliability.

### 3.3 Correlation analysis of motivation for teaching CMAs, sense of belonging, and perseverance in teaching CMAs

Table 3 presents the descriptive statistics and correlation coefficients for Motivation for Teaching CMAs, Sense of Belonging and Perseverance in Teaching CMAs. Correlation coefficients with statistical significance related to Perseverance in Teaching CMAs are highlighted in bold, with M ± SD denoting mean ± standard deviation. The bold italic numbers on the diagonal are the square roots of the Average Variance Extracted (AVE) for each variable. The results indicate a positive correlation between Perseverance in Teaching CMAs and Sense of Belonging (*r* = 0.213, *p* < 0.05), a positive correlation with Intrinsic Motivation (*r* = 0.377, *p* < 0.01), a positive correlation with Integrated Regulation (*r* = 0.427, *p* < 0.01), a positive correlation with Identified Regulation (*r* = 0.292, *p* < 0.01), a positive correlation with Introjected Regulation (*r* = 0.230, *p* < 0.01), and a negative correlation with Amotivation (*r* = −0.283, *p* < 0.01). There was no significant correlation with External Regulation. Additionally, comparing the square root of each variable’s AVE with its correlation coefficients with other variables, the square root of the AVE for each variable was greater than its correlations with other variables, indicating discriminant validity among the variables in this study.

**Table 3:** Descriptive statistics and correlation coefficients for factors (*N* = 144).

	*M* ± *SD*	(1)	(2)	(3)	(4)	(5)	(6)	(7)	(8)
(1) PiTCMA	5.777 ± 1.257	***0.719***	–	–	–	–	–	–	–
(2) Sense of belonging	6.442 ± 0.753	**0.213** ^*^	***0.809***	–	–	–	–	–	–
(3) Intrinsic motivation	6.095 ± 1.119	**0.377** ^**^	0.466^**^	***0.886***	–	–	–	–	–
(4) Integrated regulation	6.428 ± 0.924	**0.427** ^**^	0.407^**^	0.770^**^	***0.915***	–	–	–	–
(5) Identified regulation	6.137 ± 0.993	**0.292** ^**^	0.443^**^	0.686^**^	0.779^**^	***0.786***	–	–	–
(6) Introjected regulation	4.736 ± 1.787	**0.230** ^**^	0.258^**^	0.350^**^	0.332^**^	0.372^**^	***0.819***	–	–
(7) External regulation	2.889 ± 1.803	0.076	0.101	0.155	0.097	0.129	0.396^**^	***0.893***	–
(8) Amotivation	1.676 ± 1.160	**−0.283** ^**^	−0.120	−0.181^*^	−0.252^**^	−0.247^**^	0.123	0.210^*^	***0.823***

The bold italic numbers on the diagonal are the square roots of the AVE for that variable; PiTCMA, perseverance in teaching Chinese martial arts. ^*^p < 0.05, ^**^p < 0.01.

### 3.4 Structural model test of the perseverance behavior formation mechanism in teaching CMAs

#### 3.4.1 Testing the second-order model of motivation

##### 3.4.1.1 Autonomous motivation

**Figure 2:**
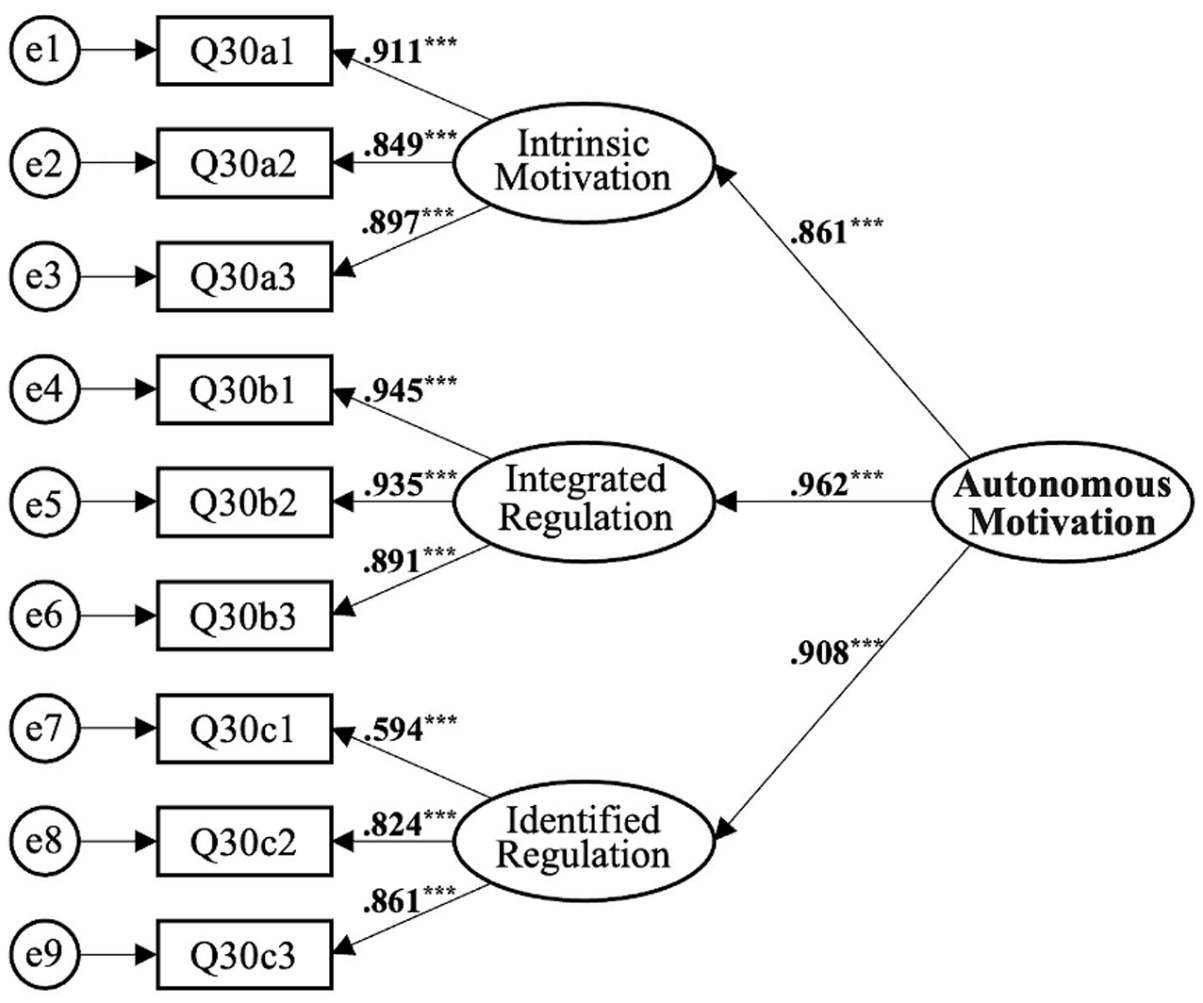
Second-order Model of Autonomous Motivation. ^***^p < 0.001.

Validation of the second-order model of autonomous motivation was conducted in three steps. First, the model’s ability to explain the first-order constructs was assessed. According to Marsh and Hocevar (1985), a target coefficient (related chi-square value of the first-order factor/chi-square value of the second-order model) close to 1 suggests a precise model. The target coefficient for autonomous motivation was found to be 1, indicating excellent adaptability of the second-order CFA indices. Second, the fit of the second-order model was assessed, revealing *χ*^2^/df = 1.184, GFI = 0.937, CFI = 0.983, and RMSEA = 0.075, indicative of a good fit (see Table 4). Third, convergent validity and composite reliability were evaluated, with AVE = 0.830 and CR = 0.936, meeting the requirements. Hence, the second-order model of autonomous motivation is deemed acceptable, adequately explaining the first-order factor constructs (see Figure 2, Table 5).

**Table 4:** Goodness-of-fit indexes for alternative models of the second-order autonomous motivation (*N* = 144).

	*χ* ^2^	df	*χ*^2^/df	GFI	AGFI	CFI	RMSEA
0. Null model	1185.613	36	32.934	0.000	0.000	0.000	0.473
1. 1 First-order factor	158.647	27	5.876	0.787	0.646	0.885	0.185
2. 3 First-order factors (uncorrelated)	313.863	27	11.625	0.701	0.502	0.750	0.273
3. 3 First-order factors (correlated)	43.530	24	1.814	0.937	0.882	0.983	0.075
4. 1 Second-order factor	43.530	24	1.814	0.937	0.882	0.983	0.075
Reference value	Smaller is better	Larger is better	<3	>0.9	>0.9	>0.9	<0.08

df, degrees of freedom; GFI, goodness of fit index; AGFI, adjusted goodness of fit index; CFI, comparative fit index; RMSEA, root mean square error of approximation.

**Table 5:** Confirmatory factor analysis for the second-order model of autonomous motivation.

Second-order factor	First-order factors	Model parameter estimates				Convergent validity			
		UFL	S.E.	*t*	*P*	SFL	SMC	C.R.	AVE
Autonomous motivation (*α* = 0.893)	Intrinsic motivation	1.000				0.861	0.741	0.936	0.830
	Integrated regulation	0.928	0.081	11.480	^***^	0.962	0.925		
	Identified regulation	0.955	0.140	6.830	^***^	0.908	0.824		

UFL, unstandardized factor loadings; S.E., standard error; SFL, standardized factor loadings; SMC, squared multiple correlations; C.R., composite reliability; AVE, average variance extracted; ^***^p < 0.001.

##### 3.4.1.2 Controlled motivation

**Figure 3:**
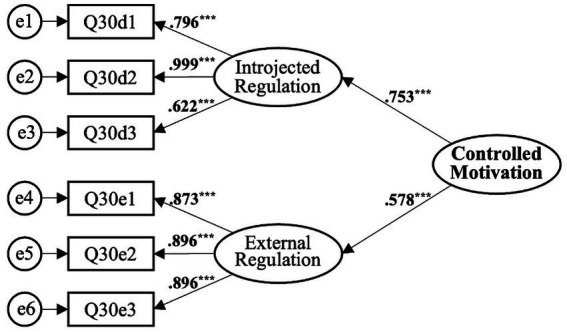
Second-order model of Controlled Motivation. ^***^p < 0.001.

Similarly, the validation of the second-order model of controlled motivation proceeded through three main steps. Initially, the model’s explanatory power for first-order constructs was tested, yielding a target coefficient of 0.89 as per Marsh and Hocevar (1985), signifying the model’s satisfactory precision. The model fit indices were exemplary, with *χ*^2^/df = 0.850, GFI = 0.983, AGFI = 0.960, CFI = 1, and RMSEA = 0.000, denoting an excellent fit (see Table 6). Convergent validity and composite reliability assessments showed AVE = 0.451 and CR = 0.617, both of which are within acceptable ranges as per Fornell and Larcker (1981a,b) criteria, thus validating the second-order model of controlled motivation (see Figure 3 and Table 7).

**Table 6:** Goodness-of-fit indexes for alternative models of the second-order controlled motivation (*N* = 144).

	*χ* ^2^	DF	*χ*^2^/df	GFI	AGFI	CFI	RMSEA
0. Null Model	570.625	15	38.042	0.421	0.189	0.000	0.509
1. 1 First-order factor	183.776	9	20.420	0.718	0.343	0.685	0.369
2. 2 First-order factors (uncorrelated)	32.567	9	3.619	0.935	0.849	0.958	0.135
3. 2 First-order factors (correlated)	6.793	8	0.849	0.985	0.960	1.000	0.000
4. 1 Second-order factor	7.651	9	0.850	0.983	0.960	1.000	0.000
Recommended values	Smaller is better	Larger is better	<3	>0.9	>0.9	>0.9	<0.08

df, degrees of freedom; GFI, goodness of fit index; AGFI, adjusted goodness of fit index; CFI, comparative fit index; RMSEA, root mean square error of approximation.

**Table 7:** Confirmatory factor analysis for the second-order model of controlled motivation.

Second-order factor	First-order factors	Model parameter estimates				Convergent validity			
		UFL	S.E.	*t* value	*P*	SFL	SMC	C.R.	AVE
Controlled motivation (*α* = 0.893)	Introjected regulation	1.000				0.753	0.567	0.617	0.451
	External regulation	0.716	0.087	8.255	^***^	0.578	0.334		

UFL, unstandardized factor loadings; S.E., standard error; SFL, standardized factor loadings; SMC, squared multiple correlations; C.R., composite reliability; AVE, average variance extracted; ^***^p < 0.001.

#### 3.4.2 Testing the formation mechanism model of perseverance in teaching CMAs

Sense of Belonging, Autonomous Motivation, Controlled Motivation, and Amotivation are treated as exogenous variables, and Perseverance in Teaching CMAs is treated as an endogenous variable. The model fitting is conducted using the Maximum Likelihood (ML) estimation method.

(1) Overall Model Fit Test. Initially, the model’s conformity to a normal distribution is tested. The skewness and kurtosis coefficients for each observed variable in this study meet the requirements of a normal distribution (Kline, 1998). Subsequently, the overall model fit is tested. The model’s overall fit is assessed based on absolute fit measures, incremental fit measures, and parsimonious fit measures. The overall fit indices for the structural model of the perseverance behavior formation mechanism in international CMAs instructors, as shown in Table 8, are as follows:

- Absolute Fit Measures: these indices evaluate how well the proposed model reproduces the observed data. The chi-square (*χ*^2^) value was noted at 140.280, where a lower value indicates a better fit. Although chi-square is sensitive to sample size, it provides a fundamental measure of model discrepancy. The Goodness-of-Fit Index (GFI) was recorded at 0.884, slightly below the recommended threshold of 0.90, suggesting a marginally acceptable fit. The Root Mean Square Error of Approximation (RMSEA) stood at 0.080, aligning with the threshold of 0.08, indicating a reasonable error of approximation by the model.
- Incremental Fit Measures: these measures compare the proposed model against a baseline model, usually a null model with no relationships among variables. The Normed Fit Index (NFI) was 0.880, and while it was just below the preferred benchmark of 0.90, it signals a moderate improvement over the baseline model. The Incremental Fit Index (IFI), Tucker-Lewis Index (TLI), and Comparative Fit Index (CFI) exhibited values of 0.938, 0.922, and 0.937, respectively, all surpassing the 0.90 mark, which indicates a substantial improvement from the baseline model and suggests a good fit.
- Parsimonious Fit Measures: aimed at evaluating the model’s fit while considering its complexity, the parsimony ratio of chi-square to degrees of freedom (*χ*^2^/df) was found to be 1.922, well below the maximum acceptable ratio of 3, denoting a good balance between model fit and parsimony.

**Table 8:** Goodness-of-fit indexes for the research model.

Fit index	Index value	Reference value	Test result
(1) Absolute fit indexes			
*χ* ^2^	140.280	Smaller is better	✓
GFI	0.884	>0.90	Nearly meets
RMSEA	0.080	<0.08	Nearly meets
(2) Incremental fit indexes			
NFI	0.880	>0.90	Nearly meets
IFI	0.938	>0.90	✓
TLI	0.922	>0.90	✓
CFI	0.937	>0.90	✓
(3) Parsimony fit index			
*χ*^2^/df	1.922	<3	✓

GFI, goodness of fit index; RMSEA, root mean square error of approximation; NFI, normed fit index; IFI, incremental fit index; TLI, Tacker-Lewis index; NNFI, non-normed fit index; CFI, comparative fit index.

In summary, despite certain indices slightly missing their respective recommended thresholds, the overall fit indices collectively indicate an acceptable level of model fit to the data, thereby supporting the structural model of perseverance behavior formation in international CMAs instructors as a viable representation of the underlying psychological processes.

**Figure 4:**
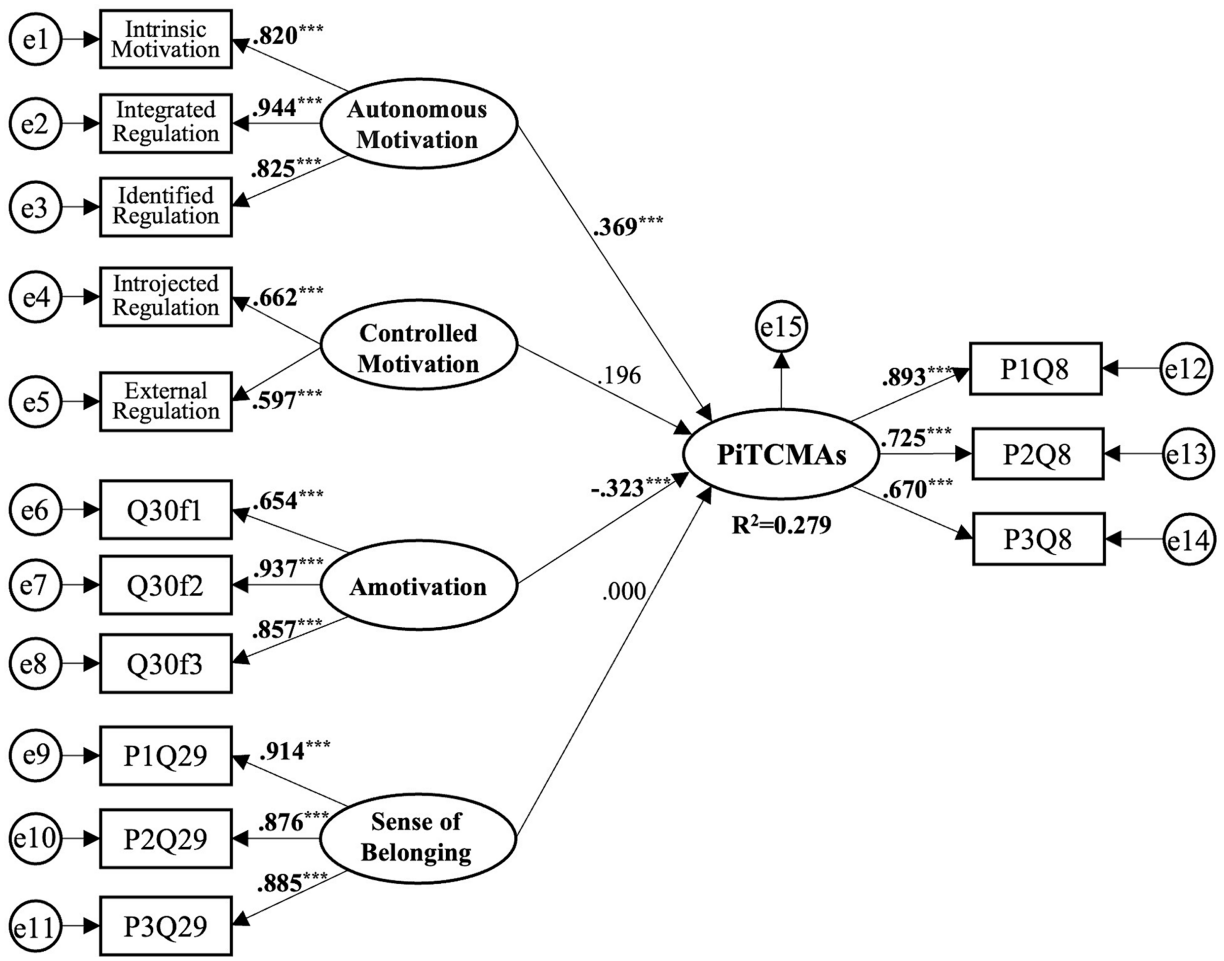
Research Model Path Diagram. Notes: PiTCMAs – Perseverance in Teaching Chinese Martial Arts. ^***^p < 0.001.

(2) Model Path Analysis and Hypothesis Testing. Figure 4 displays the structural model of the perseverance behavior formation mechanism in international CMAs instructors. The results indicate that Autonomous Motivation has a significant positive impact on Perseverance in Teaching CMAs (*β* = 0.369, *b* = 0.465, *t* = 4.232, *p* < 0.001), supporting H1; Amotivation has a significant negative impact on Perseverance in Teaching CMAs (*β* = −0.323, *b* = −0.382, *t* = −3.561, *p* < 0.001), supporting H3; Controlled Motivation has no significant impact on Perseverance in Teaching CMAs, consistent with the original hypothesis H2; Sense of Belonging has no significant impact on Perseverance in Teaching CMAs, not supporting H4; the model explains 27.9% of the variance in Perseverance in Teaching CMAs (see Table 9).

**Table 9:** Path analysis of the research model.

Paths between latent variables	*b*	S.E.	*t*	*β*	*P*	Corresponding hypothesis	Test result
PiTCMA ← Autonomous motivation	0.465	0.110	4.232	0.369	^***^	H1	✓
PiTCMA ← Controlled motivation	0.192	0.134	1.432	0.196	0.152	H2	✓
PiTCMA ← Amotivation	−0.382	0.107	−3.561	−0.323	^***^	H3	✓
PiTCMA ← Sense of belonging	0.000	0.130	0.003	0.000	0.998	H4	✕

*b*, unstandardized path coefficients, represents the direct effect of one variable on another, measured in the original units of the variables; S.E., standard error; *β*, standardized path coefficients, indicates the strength and direction of the relationship between variables, measured in standard deviation units, facilitating comparison across different paths in the model; PiTCMAs, perseverance in teaching Chinese martial arts; ^***^p < 0.001.

## 4 Discussion

This study, grounded in Self-Determination Theory, conducts an empirical exploration into the mechanisms behind the development of perseverance behaviors in the international promotion of CMAs. Our findings reveal that: (1) Autonomous motivation significantly enhances perseverance behavior; (2) Amotivation notably undermines perseverance behavior; (3) Controlled motivation does not significantly affect perseverance behavior; (4) Belongingness does not significantly influence perseverance behavior.

### 4.1 Conceptual explanation of perseverance in teaching CMAs

Perseverance in the context of teaching CMAs is characterized by the sustained efforts of international instructors to continue their educational endeavors despite encountering challenges. Our study operationalizes perseverance as a construct to evaluate the steadfastness in teaching CMAs. Through an examination of standardized factor loadings from our measurement items, we have identified the most salient indicators of perseverance, as detailed in Table 2. Primarily, these are the habitual nature of teaching CMAs (0.879) and the resolve to continue teaching against the constraints of time (0.868). These findings intimate that for those engaged in the propagation of CMAs, perseverance transcends mere volitional act, embedding itself into their daily practices and value systems.

A moderate factor loading of 0.654 emphasizes the importance of emotional investment in maintaining efforts to disseminate CMAs. This attachment may arise from a profound affinity for CMAs, gratification derived from teaching, or a sense of duty toward preserving culture. Moreover, the factor loading of 0.617 associated with the anticipation of future CMA engagements serves as a significant motivational force. This prospective enthusiasm can fortify an individual’s resolve in the face of adversity.

Additionally, a willingness to confront and navigate impediments, despite its relatively lower factor loading of 0.499, underscores the necessity for resilience and adept problem-solving in promotional endeavors. This suggests that instructors retain their zeal and determination to spread CMAs knowledge, even when confronted with challenges.

The synthesis of these measurement items and their respective factor loadings paints a multifaceted picture of perseverance in the teaching of CMAs. It encapsulates habit formation, emotional engagement, forward-looking motivation, and resilience against obstacles. Instructors who exhibit elevated levels of perseverance demonstrate a propensity for persistence-related behaviors, marked by patience, dedication to teaching CMAs, and a proactive stance in surmounting difficulties. Perseverance here is not solely about the commitment to teach; it signifies an allegiance to the conservation and proliferation of a cultural legacy. The insights gleaned from these findings are pivotal for bolstering the international propagation of CMAs, suggesting that reinforcing these key dimensions can considerably aid in their global promotion.

### 4.2 Explanation of the formation mechanism of perseverance in teaching CMAs

#### 4.2.1 The impact of autonomous motivation on perseverance in teaching CMAs

Our research findings reveal a positive correlation between autonomous motivation and perseverance in teaching CMAs, evidenced by a significant regression coefficient (*β* = 0.369, *p* < 0.001). This indicates that instructors who are autonomously motivated are more inclined to sustain and continue their teaching endeavors. This correlation is in line with the findings of Grolnick and Ryan (1987), who argued that autonomous motivation is more conducive to the endurance of behavior than other forms of motivation, including introjected regulation, external regulation, and amotivation. Deci and Ryan (2008) posit that autonomous motivation correlates with more positive behaviors, attitudes, and emotional responses, potentially accounting for its significant link to the perseverance of teaching behaviors. Empirical studies by Vallerand et al. (1997) and Black and Deci (2000) further corroborate the critical role of autonomous motivation in promoting sustained engagement in activities such as teaching CMAs.

Within the construct of autonomous motivation - comprising intrinsic motivation, integrated regulation, and identified regulation - integrated regulation emerged as the most substantial factor (standardized factor loading = 0.944), followed by identified regulation (0.825) and intrinsic motivation (0.820) (see Figure 4). Integrated regulation, characterized by a profound understanding and internalization of the behavior’s value, likely contributes most significantly to teaching perseverance. Identified regulation pertains to the recognition of a behavior’s relevance to personal goals and values. Intrinsic motivation involves participation in an activity for inherent satisfaction. Consistent with Núñez et al. (2010), our study adopts a non-discriminatory approach to these autonomous motivation types due to their continuum nature.

Instructors with high autonomous motivation not only derive enjoyment and satisfaction from teaching CMAs but also acknowledge and incorporate the value of teaching into their core beliefs and lifestyle. This integration reflects a psychological mechanism that aligns personal significance and objectives with one’s profession, as noted by Littman-Ovadia and Lavy (2016).

Our survey results revealed that respondents were autonomously motivated in their dedication to teaching CMAs. Master D.C., with over 30 years of experience teaching Tai Chi in the United States, expressed, “Tai Chi is a way of life. My life’s goal is to elevate my own Tai Chi practice while improving the health and well-being of others.” Echoing this sentiment, another master stated, “Chinese martial arts are my life. The longer I teach Tai Chi, the more I understand its significance in today’s world, which is plagued by violence and illness. If everyone could grasp the essence of Tai Chi, the world would be a more peaceful place.” Moreover, our study found that 15.0% of the CMA schools operate on a non-profit basis, with one master emphasizing, “I teach CMAs out of respect for tradition, not for profit.” This highlights how the perseverance of international instructors is often fueled by a deep alignment with personal and cultural values.

Instructors also cited the values embedded in CMAs, such as humility, respect, and justice, as key to their persistence. One instructor noted, “Learning CMAs teaches you to cultivate humility and respect, which are necessary for peace and harmony with the world. CMAs guide you to live in harmony with yourself, others, and nature, to seek peace and to defend it when necessary. They foster a sense of brotherhood and inspire one to be a person of moral integrity, standing up for justice and protecting the weak. The important values I’ve gained through CMAs – respect, perseverance, and justice – I strive to embody and share through my teaching. I believe CMAs are superior to other martial arts because their philosophy imparts a timeless essence and value. Without its virtues and historical richness, the teaching of CMAs would not fulfill its true purpose.” This reflection aligns with Howard et al. (2021), who found identified regulation to be closely linked with persistence.

In conclusion, autonomous motivation significantly influences international instructors’ perseverance in teaching CMAs. This motivation is manifested through the alignment of CMAs with personal ambitions and the embodiment of its intrinsic values. The degree to which instructors internalize and resonate with the cultural essence of CMAs correlates with their teaching tenacity. Our findings underscore the need to nurture instructors’ autonomous motivation to facilitate CMAs’ global propagation. This has implications for developing strategies aimed at bolstering teaching perseverance, suggesting that fostering autonomous motivation could be instrumental in promoting sustained instruction of CMAs.

#### 4.2.2 The impact of controlled motivation and amotivation on perseverance in teaching CMAs

Our study reveals that controlled motivation, encompassing introjected and external regulation, does not significantly influence the perseverance of instructors in teaching CMAs. Introjected regulation, driven by self-sanctioning emotions such as guilt or shame, showed no meaningful correlation with long-term commitment, aligning with Deci and Ryan (1995) assertion that the sense of worth derived from such regulation may lead to an inconsistent motivational basis for persistence. Similarly, external regulation, motivated by external rewards or the avoidance of penalties, demonstrated a minimal correlation (*r* = 0.076) with perseverance in teaching CMAs. This supports existing literature suggesting that external motivators are less effective in sustaining long-term behaviors crucial for the enduring commitment required in CMA instruction (Vlachopoulos and Karageorghis, 2005). These findings underscore the need for strategies that foster more intrinsic forms of motivation among CMA instructors, potentially leading to more sustainable teaching commitments.

Conversely, amotivation presented a substantial negative association with instructional persistence (*β* = −0.282, *p* < 0.01), consistent with the findings of Vallerand et al. (1997). Amotivation, defined as the absence of motivation, reflects a state where individuals perceive activities as meaningless or feel incompetent to perform them. This condition is detrimental in the context of CMAs dissemination, as it undermines the instructor’s likelihood of maintaining effort and engagement.

These findings suggest that the key to promoting enduring teaching of CMAs may lie in nurturing autonomous motivation, which involves personal endorsement and a sense of volition. To foster perseverance among instructors, it is imperative to address amotivation by bolstering their autonomous motivation. This involves facilitating an environment where instructors recognize the intrinsic value of their work and perceive their role in the propagation of CMAs as significant and empowering.

In conclusion, our research underscores the importance of focusing on the quality of motivation among CMAs instructors to ensure their sustained engagement. It is imperative for dissemination strategies to prioritize the enhancement of autonomous motivation and to address any elements of amotivation, rather than relying on introjected or external regulatory factors which appear to have limited impact on long-term persistence.

#### 4.2.3 The impact of sense of belonging on perseverance in teaching CMAs

Our findings revealed a notable yet complex relationship between instructors’ sense of belonging and their perseverance in teaching CMAs. Initially, a positive correlation was observed, with a correlation coefficient of 0.213 (*p* < 0.01), suggesting a significant independent relationship. However, the introduction of this variable into a Structural Equation Model (SEM) alongside other predictors resulted in a negligible factor loading of 0.005, rendering the impact of sense of belonging on perseverance statistically insignificant. This outcome deviates from our research hypothesis, prompting a deeper examination.

The diminished significance of sense of belonging in the SEM context may be attributed to two primary factors. Firstly, the presence of variables with stronger effects within the model might overshadow those with weaker effects, such as sense of belonging, particularly when these weaker variables do not substantially enhance the model’s overall explanatory power. Secondly, the instructors’ cultural background likely plays a crucial role. Sense of belonging, in this context, encompasses feelings of allegiance to and identification with the instructors’ respective CMAs schools, reflecting a tradition of master-disciple transmission and the aspiration to honor one’s lineage, a concept deeply rooted in the cultural heritage of CMAs (Chen and Wang, 2020).

Notably, a significant portion (87.8%) of the international instructors in our study are not ethnically Chinese, potentially indicating a less profound connection to the cultural heritage and sense of belonging to CMAs compared to instructors of Chinese descent. This conjecture aligns with previous research indicating the positive effects of sense of belonging and cohesion on behavioral persistence within more confined or homogeneous groups (Carron et al., 1988). However, the unique dissemination context of our study subjects—who primarily return to their home countries to promote CMAs—might further explain the observed insignificance of sense of belonging on their perseverance.

In conclusion, the influence of sense of belonging on the perseverance of international CMAs instructors emerges as a multifaceted issue. It is shaped by the interplay of model variables, cultural backgrounds, and the distinct environments of CMAs dissemination. This complexity underscores the need for further investigation into how sense of belonging affects instructors’ commitment to teaching CMAs, with a particular focus on the nuances of cultural and environmental contexts.

### 4.3 Practical implications

Our research findings offer valuable insights for the global community of CMAs schools, program designers, and policymakers. These insights can be instrumental in developing strategies to enhance the motivation and perseverance of CMAs instructors, which is crucial for the sustainable dissemination and teaching of CMAs worldwide.

Initially, for organizations and schools teaching CMAs globally, it’s critical to recognize and foster autonomous motivation among instructors. Institutions should prioritize selecting and developing instructors who not only possess deep expertise in CMAs but also embody the philosophical and cultural values integral to these arts. Enhancing instructor commitment and teaching quality necessitates providing continuous professional development opportunities that resonate with their intrinsic interests and values. Conducting workshops on integrating CMAs philosophy with teaching methods, enhancing pedagogical skills, and effective student engagement strategies can improve instructors’ motivation and effectiveness.

Furthermore, to counteract amotivation among CMAs instructors, schools and organizations should implement targeted interventions. This could involve offering feedback that acknowledges instructors’ efforts and achievements, providing counseling services to assist instructors in overcoming challenges, and fostering an inclusive environment that respects and values diversity. Tailored support mechanisms that cater to the unique needs and concerns of individual instructors can aid in preventing feelings of incompetence or disconnection from their teaching roles.

In summary, enhancing autonomous motivation and addressing amotivation are pivotal for the perseverance of CMAs instructors. By prioritizing professional development aligned with instructors’ values and providing tailored support, CMAs institutions can ensure a passionate and committed teaching faculty, thereby contributing to the art’s global propagation and sustainability.

### 4.4 Limitations and directions for future research

While this study provides new insights and theoretical contributions to understanding persistence in teaching CMAs, it also presents limitations that direct future research. Firstly, the sample selection may limit the generalizability of our findings, as the study focuses on non-ethnically Chinese CMAs instructors from Western countries. This suggests our results might mainly reflect this group’s experiences, potentially overlooking the influences of different cultural backgrounds on teaching persistence. Future research should include a wider range of instructors to broaden the findings’ applicability and explore how cultural variations affect commitment to teaching CMAs, thereby enhancing understanding of CMAs’ global dissemination. Secondly, the scales and survey methods used, while providing important initial insights, may have limitations in capturing the complex psychological dynamics related to culture. Particularly in measuring sense of belonging, existing tools might not fully reflect the profound impact of CMAs’ traditional master-disciple relationships and cultural heritage. Future research should consider employing more in-depth qualitative research methods, such as in-depth interviews or case studies, to more comprehensively understand the impact of traditional cultural factors on sense of belonging and perseverance. Lastly, while Structural Equation Modeling (SEM) serves as a powerful tool for deciphering the complex relationships among variables, its explanatory capacity is limited by the chosen variables and theoretical framework. Unconsidered variables, such as personal experiences and social support, may significantly influence the perseverance in teaching CMAs. Future research should aim to incorporate additional psychological and social variables to gain a more comprehensive understanding of the factors affecting the perseverance in teaching CMAs. In summary, although this study contributes to understanding the role of perseverance in teaching CMAs, future research must overcome these limitations to deeply explore the complex interactions among cultural, psychological, and educational dynamics to more comprehensively understand and promote the transmission and development of CMAs.

## 5 Conclusion

This study delves into the key factors influencing the sustained motivation of international instructors teaching CMAs, highlighting the pivotal role of overcoming amotivation and fostering autonomous motivation in promoting enduring teaching engagement. Compared to short-term internal or external incentives, autonomous motivation emerges as fundamentally more effective. Additionally, the study finds that a sense of belonging does not significantly impact instructors’ perseverance, potentially reflecting the diverse cultural backgrounds of the participants. The findings highlight the importance of cultivating instructors’ passion for CMAs, increasing their recognition of the cultural essence and values of CMA, and guiding instructors to integrate the dissemination of CMAs with their personal values and life goals, thereby enhancing their long-term commitment to disseminating these arts. This study not only offers practical strategies for CMAs’ global propagation but also sets the stage for future research to explore the effects of cultural backgrounds and additional psychological factors on teaching persistence. Ultimately, enhancing our understanding of these dynamics can further support the global spread and sustainable development of CMAs.

## Data availability statement

The raw data supporting the conclusions of this article will be made available by the authors, without undue reservation.

## Ethics statement

Ethical approval was not required for the studies involving humans because this study involved non-sensitive, anonymous data collection through public domain research methods, with no potential risk to participants. The studies were conducted in accordance with the local legislation and institutional requirements. Written informed consent for participation was not required from the participants or the participants’ legal guardians/next of kin in accordance with the national legislation and institutional requirements because implied consent was obtained through completion of the survey, in line with the consent statement provided at the beginning of the survey.

## Author contributions

XC: Conceptualization, Data curation, Formal analysis, Funding acquisition, Investigation, Writing – original draft, Writing – review & editing. HL: Investigation, Methodology, Writing – review & editing.
