# Supplementary material for: Factors influencing perseverance in teaching Chinese martial arts abroad: a self-determination theory perspective among international instructors
Source: Front Psychol. 2024 Apr 30;15:1391207. doi: 10.3389/fpsyg.2024.1391207 (PMC11091383; doi:10.3389/fpsyg.2024.1391207)
Supplement: Supplementary file 1 [file Table_1.DOCX]

Supplementary Material

# Supplementary Figures and Tables

## Supplementary Tables

| **Factor** | **Reference** | **Item No.** | **Item** |
| --- | --- | --- | --- |
| **Intrinsic Motivation** | Gagné et al. (2015) | Q30a_1 | Because I have fun doing my job. |
|  |  | Q30a_2 | Because what I do in my work is exciting. |
|  |  | Q30a_3 | Because the work I do is interesting. |
| **Integrated Regulation** | Tremblay et al. (2009) | Q30b_1 | Because it has become a fundamental part of who I am. |
|  |  | Q30b_2 | Because it is part of the way in which I have chosen to live my life. |
|  |  | Q30b_3 | Because this job is a part of my life. |
| **Identified Regulation** | Gagné et al. (2010) Gagné et al. (2015) | Q30c_1 | I chose this job because it allows me to reach my life goals. |
|  |  | Q30c_2 | Because this job has personal significance to me. |
|  |  | Q30c_3 | Because this job fits my personal values. |
| **Introjected Regulation** | Gagné et al. (2015) | Q30d_1 | Because I have to prove to myself that I can. |
|  |  | Q30d_2 | Because it makes me feel proud of myself. |
|  |  | Q30d_3 | Because if I don’t carry out this job, I will feel bad. |
| **External Regulation** | Tremblay et al. (2009) Gagné et al. (2010) Gagné et al. (2015) | Q30e_1 | For the income this job provides me. |
|  |  | Q30e_2 | Because this job allows me to earn money. |
|  |  | Q30e_3 | Because this job affords me a certain standard of living. |
| **Amotivation** | Fernet et al. (2008) | Q30f_1 | I don’t know, I don’t always see the relevance of carrying out this job. |
|  |  | Q30f_2 | I used to know why I was doing this job, but I don’t see the reason anymore. |
|  |  | Q30f_3 | I don’t know, sometimes I don’t see its purpose. |
| **Sense of Belonging** | Allen (2006) | Q29_1 | I feel like a part of my group.* |
|  |  | Q29_2 | Other Kung Fu brothers and sisters in my group take my opinions seriously. |
|  |  | Q29_3 | I am included in many of the group activities.* |
|  |  | Q29_4 | I can really be myself on this group. |
|  |  | Q29_5 | Other Kung Fu brothers and sisters here like me the way I am. |
|  |  | Q29_6 | People in my group are friendly to me. |
|  |  | Q29_7 | Others in the group notice when I’m good at something. |
|  |  | Q29_8 | I am treated with as much respect as others. |
|  |  | Q29_9 | People know I can perform well.* |
|  |  | Q29_10 | I feel proud of belonging to this group. |
|  |  | Q29_11 | Other Kung Fu brothers and sisters in my group respect me. |
| **Perseverance in Teaching CMAs** | Liu et al. (2011) | Q8_1 | Spreading Chinese martial arts has become my habit. |
|  |  | Q8_2 | Even though I have limited time, I never gave up spreading Chinese martial arts. |
|  |  | Q8_3 | I will feel down if I stop spreading Chinese martial arts. |
|  |  | Q8_4 | It is difficult for me to live without spreading Chinese martial arts.* |
|  |  | Q8_5 | Even though there are difficulties in spreading Chinese martial arts, I am willing to insist on spreading it. |
|  |  | Q8_6 | After every Chinese martial arts class, I eagerly anticipate the next. |
| * items removed during the measurement model fitting process. | | | |

**Supplementary Table 1.** Overview of Scale Utilization.
